# Os centrale carpi bone bruising simulating an acute scaphoid fracture

**DOI:** 10.1007/s00256-025-05063-5

**Published:** 2025-10-29

**Authors:** Hicham Bouredoucen, Pierre-Alexandre Poletti, David Branco Ferreira, Sana Boudabbous

**Affiliations:** Division of Radiology, Department of Imaging and Medical Informatics, Geneva University Hospitals, University of Geneva, Rue Gabrielle-Perret-Gentil 4, 1211 Geneva 14, Geneva, Switzerland

**Keywords:** Os centrale carpi bone bruising, Scaphoid fracture, MRI

## Abstract

Os centrale carpi is an uncommon accessory carpal bone. We report, to the best of our knowledge, the first case of imaging findings of “os centrale carpi bone bruising” simulating an acute scaphoid fracture. The various differential diagnoses of this entity are discussed. A 22-year-old man presented to our hospital with dorsolateral wrist pain following trauma. Radiography revealed a potential fracture line in the distal pole of the scaphoid and a fracture of the base of the ulnar styloid. Magnetic resonance imaging (MRI) revealed bone bruising of the os centrale carpi and a fracture of the ulnar styloid process. The objective of this case report is to present the imaging findings of os centrale carpi bone bruising, an uncommon occurrence in wrist trauma.

## Introduction

The os centrale carpi is an uncommon accessory carpal bone. When discovered, it occupies the vestigial central row of the carpus, between the scaphoid, capitate, and trapezoid [1]. It is generally asymptomatic but may cause clinical manifestations such as intermittent or persistent pain in the dorsoradial aspect of the wrist, tenderness on palpation—particularly over the scaphotrapeziotrapezoidal joint—and painful clicking, catching, or crepitus. Symptom aggravation during forced dorsiflexion or radial deviation may also occur [2].

It may be confused with an acute scaphoid fracture, a chronic fracture with scaphoid nonunion, scaphoid necrosis, or a bipartite scaphoid. The central os carpi has been observed in association with hereditary syndromes [1, 3–5]. We report a case of an os centrale carpi in a 22-year-old man with acute posttraumatic wrist pain suggestive of a scaphoid fracture at radiography. Clinical examination revealed tenderness localized to the anatomical snuffbox, pain elicited by longitudinal compression of the thumb and palpation of the scaphoid tubercle, and exacerbation of symptoms during ulnar deviation of the wrist. Subsequent MRI reveals bone bruising of the os centrale carpi bone. To the best of our knowledge, this is the first reported MRI case in the English literature of a patient presenting with post-traumatic os centrale carpi bone contusion. We present this uncommon entity, its imaging features following trauma, and the differential diagnoses.

## Case report

**Fig. 1:**
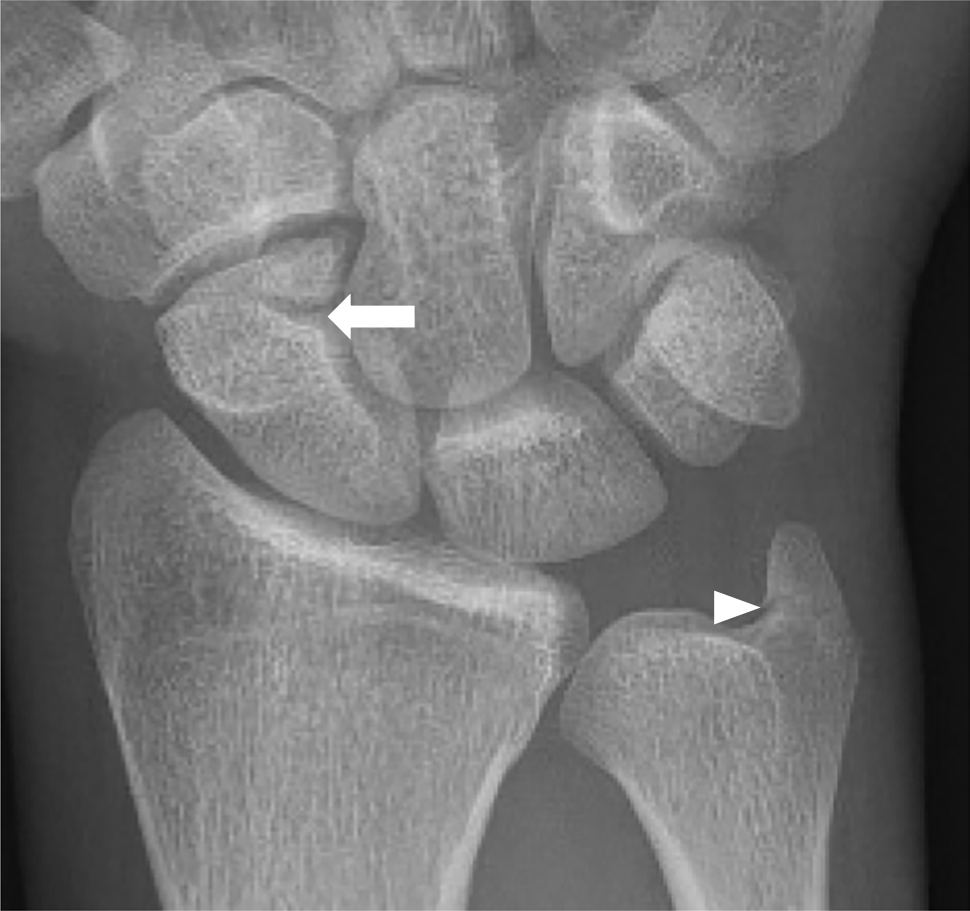
Radiography reveals a fracture of the base of the ulnar styloid (arrowhead), and a line at the distal pole of the scaphoid suggesting a fracture (arrow)

**Fig. 2:**
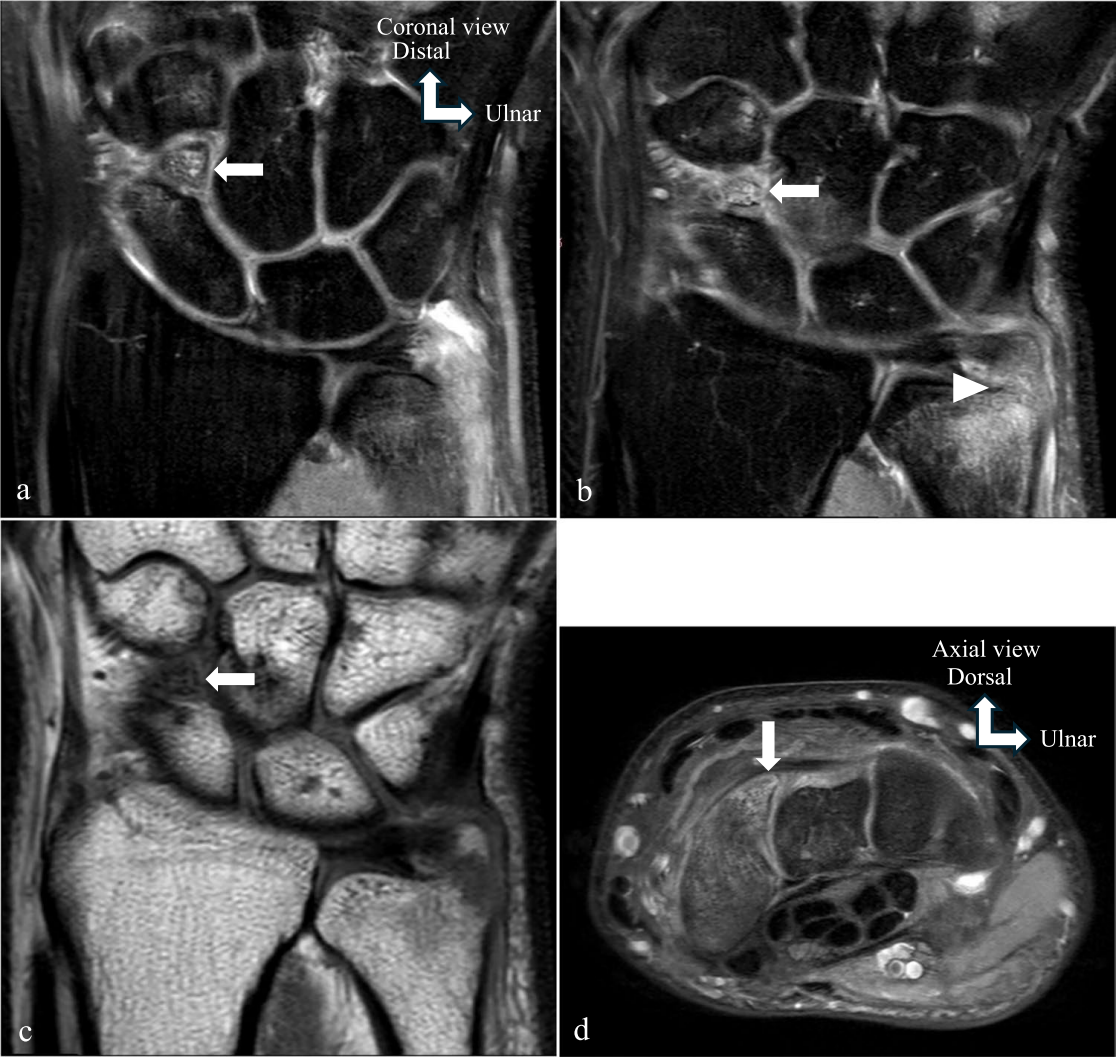
Os centrale carpi bone bruising. **a**, **b** Coronal MRI in PD FS. **c** Coronal MRI in T1-weighted TSE. **d** Axial MRI in PD FS. MRI was performed to better characterize the presumed scaphoid fracture. The MRI images confirmed a fracture of the base of the ulnar styloid (arrowhead), and also showed a triangular ossicle in the dorsal aspect of the articulation between the scaphoid, capitate, and trapezoid bones (arrows). The adjacent distal pole of the scaphoid and this ossicle presented intense edema. The presence of marked smooth sclerotic edges of this ossicle and the adjacent distal pole of the scaphoid, as well as subchondral sclerosis and fibrocartilaginous tissue on the surface between these two osseous components, called into question the diagnosis of a fracture of the distal pole of the scaphoid and confirmed os centrale carpi bone bruising

A 22-year-old patient presented following a forced twisting injury of the wrist. He reported operating a heavy impact wrench that suddenly jammed and abruptly restarted, resulting in an accidental episode of excessive supination combined with dorsal extension of the wrist. Physical examination revealed pain in the distal end of the ulna and the dorsolateral aspect of the midcarpal joint. Posteroanterior and lateral radiographs revealed a fracture of the base of the ulnar styloid and a possible fracture line in the distal pole of the scaphoid (Fig. 1). The radiographic appearance suggested a chronic fracture due to sclerosis of the fracture margins. The radiographic appearance was more indicative of chronic mechanical impingement between the distal pole of the scaphoid and the distal ossicle. An MRI was performed to distinguish an acute fracture from a pre-existing scaphoid lesion resulting from a prior trauma, with secondary progression to osseous nonunion. The MRI (Fig. 2) confirmed, in addition to the fracture of the ulnar styloid process, the presence of a small triangular ossicle located on the dorsal aspect of the joint between the scaphoid, capitate, and trapezoid bones, as well as intense bone edema of the distal pole of the scaphoid and this ossicle. However, the presence of smooth, well-defined sclerotic edges of the ossicle, its immediate proximity to the distal pole of the scaphoid, along with subchondral sclerosis and the presence of fibrocartilaginous tissue at the interface between these two osseous structures, allowed the exclusion of the diagnosis of an acute scaphoid fracture. The presence of these signs of stress response and degeneration may suggest pseudarthrosis. Scaphoid pseudarthrosis occurs in about 36% of proximal fractures, 20% of waist fractures, and 0% of distal fractures [6]. An uncommon anatomical variant was also suspected. A radiograph performed 1 year earlier revealed the same feature. The final diagnosis was os centrale carpi bone bruising, based on the presence of bone marrow edema and the absence of a hypointense fracture line. This was associated with a fracture of the base of the ulnar styloid. After 3 months of conservative treatment, the patient has complete alleviation of his symptoms with a return to his usual activities.

## Discussion

The os centrale carpi is an uncommon accessory carpal bone. Gruber found 22 cases of persistent central carpal bone in adults among 5292 carpals studied [7]. It is located on the dorsal surface of the carpal bones, interposed in the space between the scaphoid, capitate, and trapezoid bones [1], constituting a component of the midcarpal complex of the wrist. It may be partially united to the scaphoid [8]. The os centrale carpi is commonly present in many mammals but is generally absent in humans and great apes, including gorillas and chimpanzees [9]. Phylogenetically, the os centrale carpi is a remnant of the central row of the carpal bones. The cartilaginous os centrale carpi is generally present in the developing human embryo and fetus around the sixth week of gestation [10]. It usually fuses with the radial carpal bone towards the end of the second or third month of intrauterine life to form the scaphoid. Its incorporation into the scaphoid is represented by a prominent tubercle on the dorsomedial edge of the scaphoid or by a notch on this edge that leads to a biscuit shape [9]. In some cases, the central bone may fuse with the capitate or trapezium [4]. Three types of radiological presentation of the central carpi bone are possible [1, 5]: (1) a well-defined, independent bone, single or double, (2) an incompletely separated osseous element with smooth contours, or (3) an empty radiographic space between the scaphoid, capitate, and trapezoid filled with synovial tissue [1]. The differential diagnosis, in the case of an isolated finding, includes acute scaphoid fracture, chronic scaphoid fracture with non-union, scaphoid necrosis, and bipartite scaphoid [1, 3]. In acute scaphoid fractures, the edges are sharp or irregular but non-corticated, and the presence of a well-defined cortical margin generally aids in diagnosis. The os centrale carpi can be mistaken for a chronic fracture with non-union. A chronic fracture [11, 12] may present with diastasis between the proximal and distal fragments, cystic changes, or marked bone resorption along the fracture line. The distal fragment may show osteopenia, whereas the proximal fragment may exhibit variable, normal, or increased density. In the absence of a history of trauma, radiographs may fail to distinguish a central carpal bone from a chronic scaphoid fracture [1]. A bipartite scaphoid is another differential diagnosis [1, 3]. It consists of two fragments forming the scaphoid, separated by a transverse radiolucent line located at the proximal pole rather than the distal pole. When combined volumetrically, the two fragments of a bipartite scaphoid form a normal complete scaphoid, whereas a central carpal bone occupies additional space compared to a complete scaphoid [3, 13]. The os centrale carpi has also been associated with certain congenital malformations, including Holt-Oram syndrome, Larsen syndrome, oto-palato-digital syndrome, and hand-foot-uterus syndrome, among others [1, 3–5]. Outside of these syndromes, this bone is unusual in isolation and rarely bilaterally [3, 14]. The os centrale carpi is usually asymptomatic. However, this bone is recognized as a cause of pain [11]. Patients with symptomatic os centrale carpi may present with chronic wrist pain, tenderness on palpation of the dorsum of the hand or wrist radiating to the thenar eminence, with or without swelling. The pain may be associated with a clicking sound during forced supination/pronation or during wrist extension and radial deviation [11]. The os centrale carpi may present with intermittent wrist pain due to ossicular mobility interfering with the movement of the surrounding carpal bones, probably after post-traumatic ossicular detachment [2], or following osteonecrosis of a central carpal bone that causes secondary degenerative arthritis and synovitis [14]. Osteonecrosis of the central carpal bone presents with a fragmented and sclerotic appearance, joint irregularity, and narrowing [3]. It is best assessed by MRI, particularly with dynamic sequences following intravenous gadolinium injection, which allows precise evaluation of bone perfusion. Although necrotic bone may sometimes show enhancement due to fibrovascular ingrowth, dynamic contrast-enhanced MRI may be used to determine the degree of ischemia and the extent of necrosis [15]. The exact origin of osteonecrosis is not clearly identified; one hypothesis has been proposed: an ossicle initially partially attached to the scaphoid becomes detached following trauma, and the vascular supply from the scaphoid is interrupted, leading to osteonecrosis of the os centrale carpi [14]. If an os centrale carpi is symptomatic, it can be excised [2, 11]. Os centrale carpi bone bruising occurred as a result of the forced twisting mechanism combined with dorsal extension of the wrist. In addition, during a forceful hypersupination movement, fractures of the ulnar styloid primarily result from excessive traction exerted by the distal radioulnar ligaments and the fibrous subsheath of the extensor carpi ulnaris tendon, both of which are firmly attached to this osseous structure. This tensile stress leads to an avulsion fracture of the ulnar styloid. The mechanism is further exacerbated by excessive rotational force, which imposes significant strain on these stabilizing structures anchored to the ulnar styloid [16].

## Conclusion

Os centrale carpi is an uncommon anatomical variant which can be specifically injured. Radiographs and MRI help best depict this injury and distinguish it from a distal scaphoid fracture.

## Data Availability

Not applicable.
